# Role of Salinity and Ionic Composition on the Kinetic Stability of Water-in-Oil Model Emulsions

**DOI:** 10.1021/acsomega.6c02106

**Published:** 2026-08-01

**Authors:** Julia M. Castro, Rayane R. B. Corona, Rogério Ramos, Cleocir J. Dalmaschio, Cristina M. S. Sad, Eustaquio V. R. Castro

**Affiliations:** † NEMOG, Mechanical Engineering Department, Federal University of Espírito Santo, Av. Fernando Ferrari, 514, Goiabeiras, Vitória 29075-073, Espírito Santo, Brazil; ‡ LABPETRO, Chemistry Department, 28126 Federal University of Espírito Santo (UFES), Av. Fernando Ferrari, 514, Goiabeiras, Vitória 29075-073, Espírito Santo, Brazil

## Abstract

The formation of water-in-oil (W/O) emulsions represents a significant economic and operational challenge in the petroleum industry. In this study, the effects of aqueous-phase salinity on the kinetic stability of W/O model emulsions were investigated using a medium-density lubricating oil, the nonionic surfactant Triton X-114, and aqueous phases with varying ionic compositions, including deionized water, tap water, produced water, and saline solutions ranging from 35 to 500 g L^–1^, with suspended solids present at concentrations above the saturation limit. The results revealed that stability is nonlinear with respect to salinity. Emulsions with intermediate ionic strength exhibited enhanced performance. In particular, the emulsion prepared with produced water had the smallest droplets and maintained kinetic stability for up to 24 h under gravitational conditions. The emulsion prepared with 35 g L^–1^ NaCl also exhibited a significant reduction in droplet size. This behavior is attributed to salting-in effects and ion–dipole interactions that promote denser interfacial packing. In contrast, highly saline systems (saturated solution and saline aqueous suspension), despite exhibiting lower interfacial tension, showed increased flocculation and phase separation because of surfactant dehydration and interfacial film weakening. Thus, it is possible to conclude that an optimal ionic strength range exists that favors interfacial structuring and that the use of model fluids is a viable and safe alternative for simulating complex petroleum interfacial phenomena at the laboratory scale.

## Graphical abstract

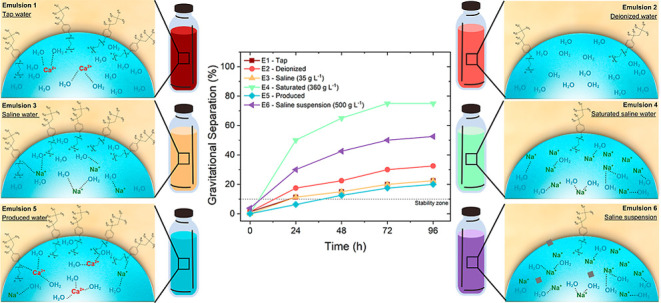

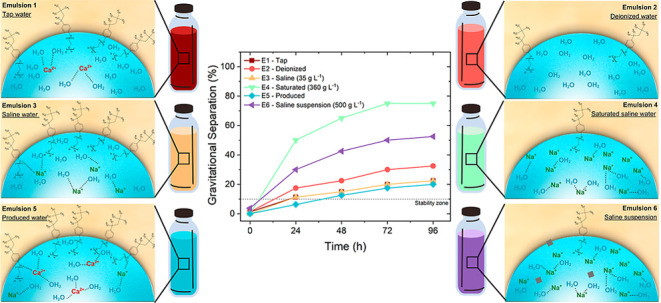

## 1 Introduction

The formation of emulsions in the oil and gas industry is a significant economic challenge. This issue is reflected not only in the costs associated with the chemicals used but also in production losses due to increased oil viscosity and corrosion in pipelines and equipment.^1,2^ In addition, pumping costs, the risks associated with erosion in pipelines and problems such as scaling and scaling contribute to the complexity of the scenario owing to the financial impacts caused.^2,3^ The consequences of crude oil emulsion formation for the industry are diverse and include the degradation of oil quality, catalyst poisoning in subsequent refining processes, and risks of environmental contamination.^4,5^ Furthermore, the water-induced viscosity increase in these systems can result in high pressure drops, which not only increase production and transport costs but also reduce crude oil throughput.^5^

During oil flow to the surface, the crude oil comes into contact with water, and despite initially being in separate phases, the turbulent flow within the pipelines generates sufficient kinetic energy to deform, break up, and disperse one phase into the other.^6^ This turbulence, produced in components such as pumps, valves, chokes, and risers, creates shear forces that disperse the water into small droplets within the oil phase, promoting the formation of stable emulsions.^6–8^ In addition to this mechanically induced mechanism, the presence of natural emulsifiers, such as asphaltenes and resins, can promote the spontaneous formation of water-in-oil (W/O) emulsions, as occurs, for example, during offshore oil production.^9,10^

When petroleum reaches surface facilities, it is accompanied by associated water originating either from geological formations or from secondary recovery processes involving water injection.^2^ This water, referred to as produced water, is typically characterized by high salinity. In the early stages of production, the volumetric fraction of produced water relative to the oil volume generally ranges from 0.5–10%. Under specific operational conditions, this fraction may increase to approximately 50%, and as a well approaches the end of its productive life, the water cut can rise substantially, in some cases approaching nearly 100%.^11–13^

In view of this, the most appropriate way to address the challenges related to emulsion formation during crude oil production involves conducting laboratory experiments that consider the temperatures and water cuts predicted for the field.^14^ Understanding the interfacial phenomena in the stabilization and destabilization processes of the water droplets in the oil emulsions during their production would allow the prevention of their behavior in the field, the improvement of the recovery factor, and the reduction of the costs related to operation. In this context, considering that the separation of W/O petroleum emulsions requires considerable amounts of energy, large physical space and several chemical products, reducing the emulsification process would result in a significant reduction in costs for the petrochemical industry.^15,16^ For this purpose, a fluid with properties similar to those of petroleum and its emulsions can be developed to simulate the flow of oil during processing in an environment different from the producing field.^16,17^ In this context, Kempin et al.^18^ developed a laboratory-scale flow loop designed to maintain dynamic similarity with real production lines. The methodology is based on adjusting the system velocity using the Weber number and integrating variables such as density, interfacial tension, and the representative droplet diameter. This approach allows model emulsions to mimic the behavior of crude oil, enabling accurate analysis of droplet breakup throughout the production process. Therefore, the advantages of using these fluids are their composition, greater economy and greater environmental and laboratory safety, depending on the fluid being simulated. This application is viable because, for the petrochemical industry, working with crude oils and their emulsions is challenging. This difficulty is related to the complex composition of petroleum, physical properties and characteristics such as toxicity, flammability and volatility.^17,19,20^

Several studies involving the use of model emulsions to investigate the behavior of real petroleum emulsions have been reported.^21–23^ Naccache et al.^24^ developed model emulsions to verify the efficiency of gravitational settlers, with the objective of replacing petroleum emulsions in tests. The authors indicated that the model emulsions showed an acceptable correspondence with the parameters established by the research group. Corona et al.^16^ evaluated the similarity of the physicochemical properties of five different mineral oils with those of petroleum, including their spectroscopic properties via mid-infrared absorption. The results indicated that the properties of the lubricating oil were similar to those of the other samples evaluated, suggesting that the lubricating oil can be used to prepare a model fluid in stable W/O emulsions. The authors obtained stable W/O emulsions with a nonionic surfactant and an aqueous phase with a salinity of 35 g L^–1^.

In thiscontext, on the basis of scientific studies, the similarity of the physicochemical, rheological and spectroscopic properties of a reference oil with those of petroleum allows the production of stable W/O emulsions for academic and industrial investigations. However, the influence of salinity on the stability of W/O emulsions remains controversial. Shams et aal.^25^ and Wang et al.^26^ reported that increasing the salinity of the aqueous phase resulted in reduced emulsion stability, favoring coalescence and phase separation. In contrast, Liu et al.^27^ and Moradi et al.^28^ reported a stabilizing effect under conditions of higher ionic strength, with an increased presence of surfactants at the interface and smaller droplets formed during emulsification, respectively. These seemingly conflicting results suggest that the role of salinity is not straightforward and may depend on factors such as the droplet size distribution, interfacial film properties, and ionic composition. Additionally, Shafiei et al.,^29^ when evaluating the type and concentration of ions in the aqueous phase of petroleum emulsions, reported that at concentrations up to 10 g L^–1^, cations stabilize the emulsions by reducing the droplet size, with divalent cations exhibiting superior performance compared to monovalent cations.

A comprehensive understanding of the influence of salinity on water–oil (W/O) model emulsions is needed to elucidate the physicochemical mechanisms governing emulsion stability and destabilization. Since the salinity and ionic composition of produced water can vary significantly among reservoirs, the use of model emulsions with controlled aqueous phases is essential for realistically reproducing petroleum-like systems. Accordingly, this study aims to systematically evaluate the effect of aqueous-phase salinity on the kinetic stability of W/O model oil-like emulsions by correlating gravitational separation, droplet size distribution, interfacial tension, and microstructural features obtained by optical microscopy. The resulting model emulsions provide a controlled platform for further studies while minimizing the operational and safety constraints associated with crude oil. Furthermore, the formulation of a simultaneously stable and representative system constitutes a fundamental step toward enabling its future application as a working fluid in a pilot-scale flow loop, allowing for a transition from fundamental interfacial characterization to the investigation of realistic hydrodynamic conditions.

## 2 Methodology

To study the effect of salinity on the stability of W/O model emulsions, a medium lubricating oil previously studied and characterized by Corona et al. was used.^16^ Initially, the oil phase and the six aqueous phases of the emulsions were prepared and analyzed in terms of specific gravity. The W/O emulsions prepared with different salinity contents were analyzed for interfacial tension, gravitational separation, droplet size distribution and optical microscopy.

### 2.1 Preparation and Characterization of the Oil Phase and Aqueous Phases

The selection of the model oil to compose the oil phase of the emulsions should be based on the similarity of the physicochemical properties of this oil with those of a medium oil.^16^ These properties include specific gravity (ASTM D5002),^30^ °API (ASTM D1250-08 and ISO 12185:1996),^31,32^ dynamic viscosity (ASTM D7042),^33^ and total acidity number (ASTM D664).^34^ The medium lubricating material was identified as the total acidity number (acidic oil is considered greater than 0.5 mg KOH g^–1^ in the petrochemical industry).^35^ The characterized physicochemical properties are listed in Table 1.

**Table 1:** Physicochemical Characterization of the Reference Oil and Medium Lubricant Used in the Model Fluid.^16^ ^,^ a

oil	specific mass (g mL^–1^) a 20 °C	°API	total acidity number (mg KOH g^–1^)
reference oil	0.8885 (0.0002)	27.1 (0.1)	0.280 (0.049)
medium lubricating oil	0.8743 (0.0001)	29.6 (0.1)	0.605 (0.011)

a Adapted with permission from Corona et al. *Geoenergy Science and Engineering*. **2023**, *221*, 111265. Copyright 2023 Elsevier.

Aqueous phases have different salinities, and their effects on the stability and homogeneity of emulsions can be evaluated. For this purpose, tap water, deionized water, saltwater (35 g L^–1^ NaCl), saturated saltwater (360 g L^–1^ NaCl), and saline aqueous suspension (500 g L^–1^ NaCl) were used. The concentration of NaCl in saltwater (35 g L^–1^ NaCl) reflects the average salinity of seawater, where in 1 L, there are approximately 35 g of dissolved salts, such as Na^+^, Ca^2+^, Mg^2+^, Cl^–^ and SO_4_2^–^.^36,37^ Tap water was titrated with EDTA (0.01 mol L^–1^) at pH 10 (with NH_4_Cl/NH_4_OH buffer) to determine the concentration of Ca^2+^ ions, following the methodology described in Harris (2017).^38^ The produced water was characterized as described by Sad,^39^ with the concentrations of the cations indicated in Table 2.

**Table 2:** Concentration of Cations in Produced Water Determined by ICP–OES

cations	produced water (mg L^–1^)
Ba^2+^	125 (4)
Ca^2+^	2787 (95)
Fe^3+^	<0.1
K ^+^	959 (25)
Mg^2+^	1025 (25)
Mn^2+^	<0.1
Na^+^	41473 (957)
Ni^2+^	0.949 (0.026)
Sr^2+^	381 (12)
V^2+^	<0.1

#### 2.1.1 Specific Mass

The specific gravity of the oil and aqueous phases was determined via a density and sound velocity meter manufactured by Anton Paar, model DSA 5000 M. The samples were inserted into the glass ampule inside the equipment using a syringe. The measurement was performed with a temperature ramp from 20 to 40 °C, with an increment of 5 °C, after the equipment checked the specific mass of the air and deionized water.

### 2.2 Preparation and Characterization of W/O Model Emulsions

The emulsions were prepared with a total volume of 40 mL, of which 90% corresponded to the continuous oil phase. A volume of 36.0 mL was measured by mass considering the density of the lubricating oil. The oil phase of the emulsions consisted of a mixture of lubricating oil with 0.1% (v/v) nonionic surfactant Triton X-114 (TX-114). The selection of TX-114 as a single emulsifying agent was intended to isolate the effect of aqueous-phase salinity on emulsion stability, thereby avoiding additional variables that could hinder the interpretation of interfacial phenomena. Although the hydrophilic–lipophilic balance (HLB) of TX-114 is 12.4, which is typically considered ideal for oil-in-water (O/W) emulsions, previous studies from our research group^40^ have demonstrated that W/O emulsions can be effectively stabilized using high-HLB surfactants such as TX-114. This behavior can be attributed to factors beyond the HLB value, considering the characteristics of the system as a whole. Thus, approximately 33.339 g of medium lubricating oil (99.9% of 36 mL) and 0.03808 g of TX-114 (0.1% of 36 mL) were weighed for each emulsion, considering ρ_oil_ = 0.8714 g mL^–1^ and ρ_TX‑114_ = 1.058 g mL^–1^, at 25 °C.

For the homogenization of the emulsions, agitation was applied at 3000 rpm for 3 min in a mechanical shaker from the manufacturer IKA, Ultra Turrax model T25, with an S25N-18G dispersing tool. This shear energy is sufficient to mimic field processing, maintaining a stable emulsion with droplet sizes smaller than 10 μm for up to 96 h or more.^16^ Each emulsion was subsequently transferred to calibrated, graduated conical glass tubes with a capacity of 100 mL.

#### 2.2.1 Effects of Salinity on Gravitational Separation

The stability of the W/O model emulsions was evaluated using a bottle test. The emulsions were kept at an ambient temperature of 25 ± 3 °C for 4 days (96 h) under visual monitoring. For evaluation, 40 mL of each model emulsion was inserted into conical glass tubes and monitored at time intervals of 24 h. At each time interval, the water separation volume was defined.

#### 2.2.2 Effect of Salinity on Droplet Size

The determination of the droplet size of each emulsion was performed using laser diffraction via a Bettersizer instrument (manufacturer AcilWeber, model ST). The mean chosen to represent the distribution data is *D*,^3,4^ a measure of the volume-weighted mean diameter, i.e., the diameter of a sphere having the same volume as the particle under consideration, which is particularly suitable for emulsion droplet size analysis.^41^ Analytical-grade isoparaffin acquired from Pochteca Coremal was used as the dispersing medium.

#### 2.2.3 Effect of Salinity on Interfacial Tension

The interfacial tension of the oil phase with the aqueous phases was measured via the Pendent drop method via the tensiometer manufacturer SEO, model Phoenix MT (M). For the measurements, a plastic syringe with a “U”-shaped needle was used, which was filled with the fluid with the lowest specific gravity. After the syringe was filled with the oil phase, it was placed on the manual piston of the equipment. The needle is immersed in a transparent container (cuvette) containing each aqueous phase (denser fluid). After the needle diameter calibration, the drop was dispensed and recorded with the aid of a camera attached to the tensiometer. After the droplet stabilized, the interfacial tension between the oil phase and the different aqueous phases was measured. This procedure was performed in triplicate at an ambient temperature of 25 ± 2 °C. The Pendent drop method is based on the Bashforth–Adams equation, which relates the droplet shape geometry to the interfacial tension.^42,43^ Images that did not present parameters *a* and *b* within the limits of the droplet were disregarded, and the reported values correspond to the average of the valid measurements.

#### 2.2.4 Effects of Salinity on Morphology

Photomicrographs of the samples were used to analyze the morphology of the droplets in the emulsions, that is, the shape of the water droplets in the oil and their coalescence. The method involved the preparation of glass slides for light microscopy, with isopropyl alcohol (PA) used as a cleaning agent. One drop of each W/O emulsion was subsequently placed on the slide. A coverslip was placed over the emulsion droplet to form a thick film that allowed the droplets to be visualized without overlapping. The images were acquired using a polarized light microscope manufactured by Nexcope, model NP900TRF, with a CFI LU Plan Fluor EPI P 20× objective and a PrimeCam 12 digital camera.

## 3 Results and Discussion

The prepared emulsions were milky and slightly yellowish in color, as shown in Figure 1. This optical behavior arises from light scattering by dispersed water droplets within the continuous oil phase. When incident light interacts with a colloidal dispersion, some of the radiation is scattered because of differences in the refractive index between the phases, resulting in the turbidity characteristic of emulsified systems.^44^

**Figure 1:**
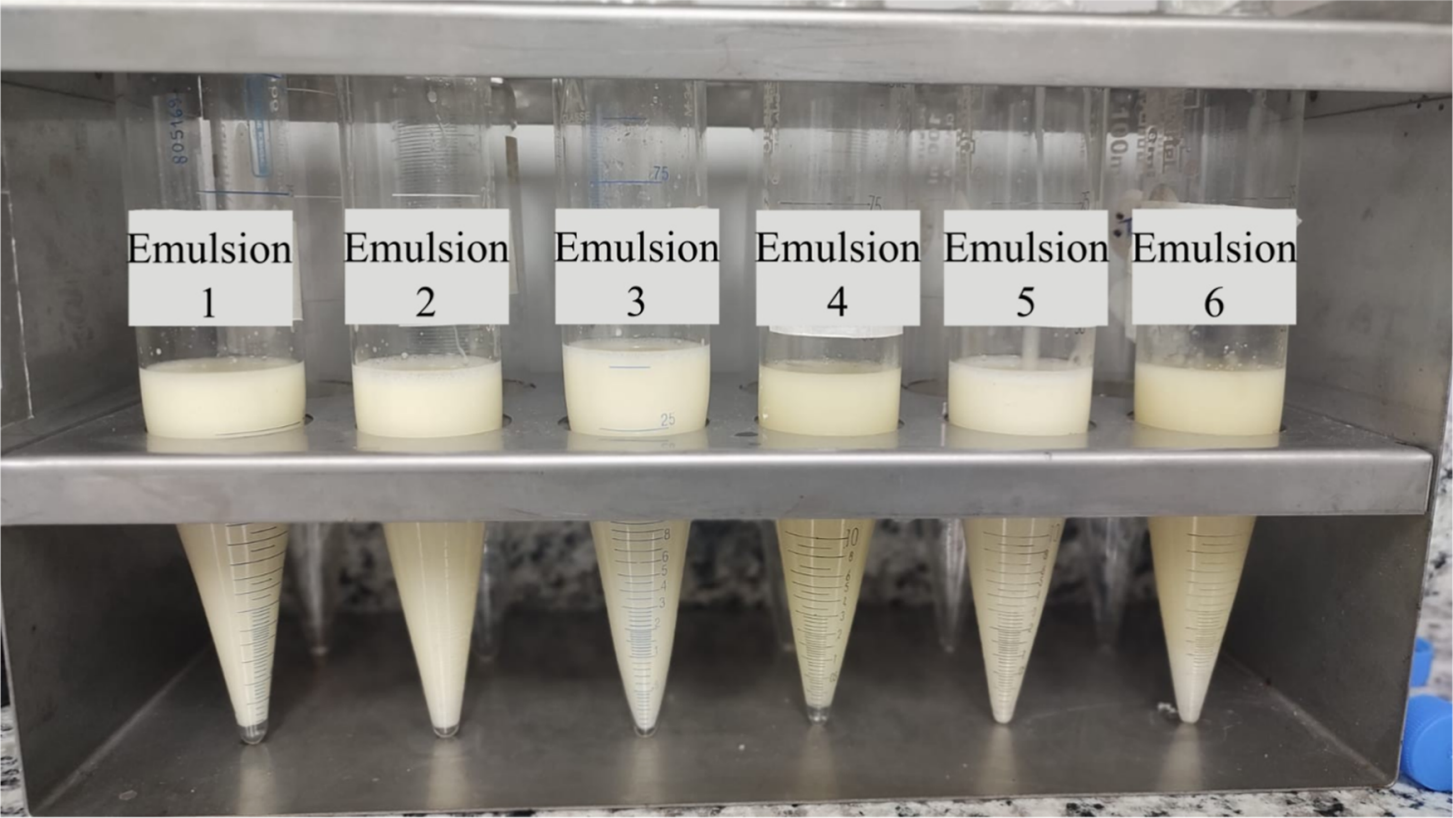
Model emulsions prepared with the following water types: (1) tap water, (2) deionized water, (3) saline solution (35 g L^–1^ NaCl), (4) saturated saline solution (360 g L^–1^ NaCl), (5) produced water (55 g L^–1^ NaCl), and (6) saline aqueous suspension (500 g L^–1^ NaCl).

After emulsification and transfer to a conical tube, Emulsion 6 showed NaCl crystallization, with solid aggregates sedimenting at the bottom of the vessel. Over time, additional salt precipitation occurred concomitantly with aqueous phase separation (Figure 2). These observations indicate that the salt concentration exceeded the solubility limit under the experimental conditions, leading to bulk crystallization. With respect to larger crystalline aggregate sediment, the possible formation of fine salt particulates cannot be excluded, and such particles may contribute to interfacial stabilization mechanisms.

**Figure 2:**
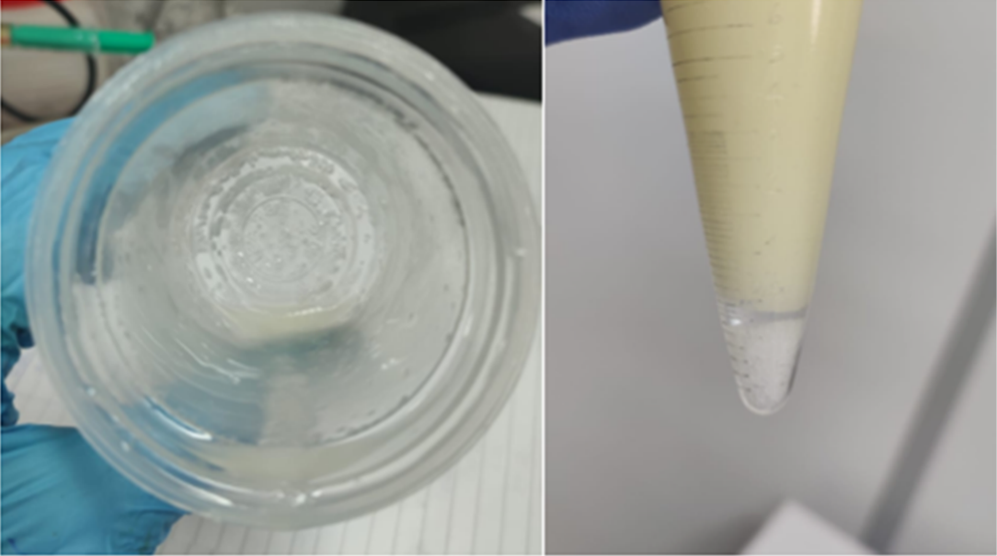
Conical glass tube containing Emulsion 6 (saline aqueous suspension, 500 g L^–1^ NaCl). Visible crystalline NaCl solids formed because of supersaturation and sedimented at the bottom of the tube, indicating bulk salt precipitation under the experimental conditions.

### 3.1 Effects of Salinity on Gravitational Separation

The difference in specific gravity, at 25 °C, between the oil phase and each aqueous phase with different salinities studied in this work is presented in Table 3. An increase in salinity results in an increase in the specific mass of the aqueous phase. The difference in specific gravity between the phases is a crucial factor for the destabilization of emulsions, as is the dynamic viscosity. Studies in the literature^45,46^ indicate that phenomena such as creaming and sedimentation are not observed in emulsions when the density difference between the phases is less than 10%. Note that all the emulsions separated the aqueous phase because of the difference in density; once in all the systems, the density of the aqueous phase was at least 10% greater than that of the oil phase (Figure 3).

**Table 3:** Differences between the Specific Gravities of the Oil Phase and Each Aqueous Phase at 25 °C

aqueous phase	difference in specific gravity and oil phase at 25 °C (%)
tap water	12.40
deionized water	12.40
saline water (35 g L^–1^ NaCl)	14.49
saturated saline water (360 g L^–1^ NaCl)	26.82
produced water (55 g L^–1^ NaCl)	15.15
saline aqueous suspension (500 g L^–1^ NaCl)	28.65

**Figure 3:**
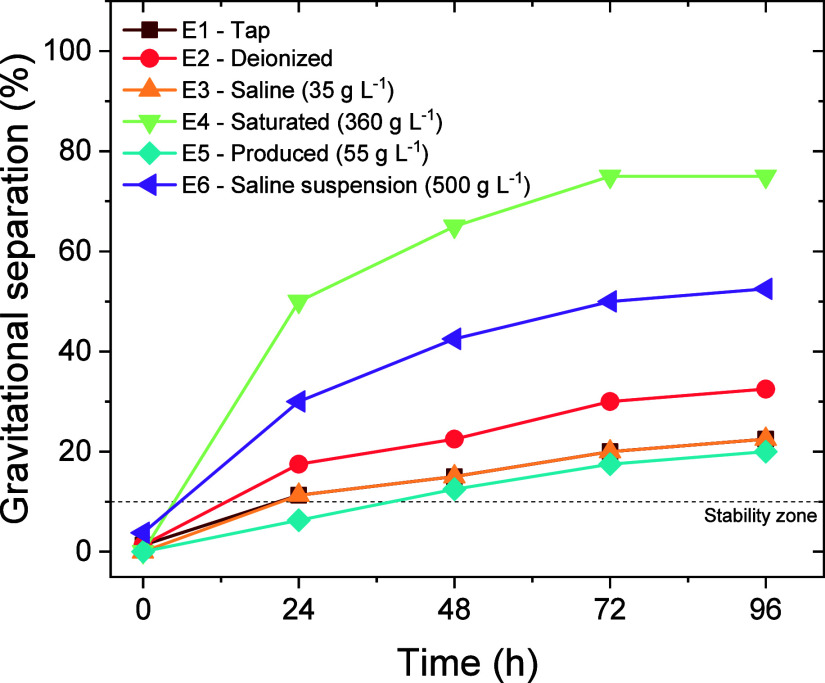
Time-dependent gravitational separation of W/O emulsions prepared with medium lubricating oil containing 0.1% (v/v) Triton X-114 and 10% (v/v) aqueous phase of varying salinity.

All the emulsions exhibited aqueous phase separation, which is consistent with the density contrast between phases, as the aqueous phase density exceeded that of the oil phase by more than 10% in all the systems. The emulsions prepared with saturated saline water (360 g L^–1^ NaCl) and saline aqueous suspension (500 g L^–1^ NaCl) presented the greatest density differences (26.82% and 28.65%, respectively) and, consequently, the most pronounced gravitational separation over time. The emulsions prepared with produced water (55 g L^–1^ NaCl), tap water, and 35 g L^–1^ NaCl solution exhibited lower phase separation than those formulated with deionized water did. This behavior suggests that moderate ionic strength and the presence of dissolved cations may influence interfacial film organization, potentially enhancing surfactant packing and modifying droplet–droplet interaction forces, thereby delaying gravitational separation. The stability threshold adopted in this study considers emulsions presenting up to 10% (v/v) separated aqueous phase relative to the initial dispersed volume as kinetically stable.^16^ According to these criteria, only the emulsion prepared with produced water maintained kinetic stability for up to 24 h under gravitational conditions.

Although the density contrast between phases contributes to gravitational instability, it does not solely determine emulsion stability. Previous studies have demonstrated that sufficiently intense homogenization conditions can yield kinetically stable W/O emulsions even when the density difference between phases exceeds 10%.^16,24^ This technique suggested that the emulsions did not show kinetic stability at *t* ≥ 96 h because of the 3 min stirring time and rotation at 3000 rpm, since they are determining factors for emulsification. Ghannan^47^ and Corona et al.^16^ reported that the improved stability of W/O emulsions is attributed to higher mixing speeds and higher concentrations of surfactants. Thus, the W/O emulsions would most likely be stable for more days if they were shaken at higher time intervals and/or speeds or had higher concentrations of surfactants. However, in this study, we chose to use the methodology normally used for petroleum emulsification, which, owing to its high emulsification of natural surfactants (resins and asphaltenes), does not require such agitation compared with model fluids.^9,48^

### 3.2 Effect of Salinity on Droplet Size

The evolution of the volume-weighted mean droplet diameter (*D* ^3,4^ ) exhibited a consistent trend across the emulsions: a decrease in the measured average droplet size with increasing stability time. This is attributed to gravitational separation processes, including sedimentation and coalescence of larger droplets, which progressively separate from the emulsion and form a free aqueous phase. As a result, the remaining emulsified fraction becomes enriched in smaller droplets, leading to a shift in the measured size distribution toward lower diameters. The results are presented in Figure 4.

**Figure 4:**
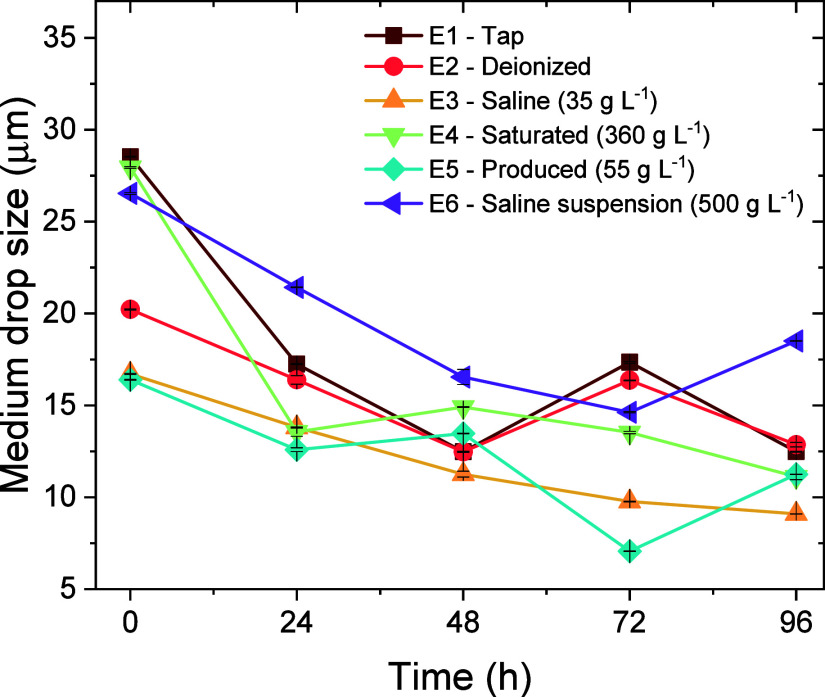
Average droplet size of W/O model emulsions prepared with different aqueous phases.

To correlate the results of the mean droplet size as a function of time together with the phase separation, the concentrations of Na^+^ and Ca^2+^ ions are presented in Table 4. The concentration of Na^+^ ions in saline aqueous suspension takes into account the solubility of NaCl, where one part is dissociated and the other is precipitated.

**Table 4:** Concentrations of the Cations Na^+^ and Ca^2+^ in the Aqueous Phases of the Emulsions Studied

		concentration (g L^–1^)	
emulsion	aqueous phase	Na^+^	Ca^2+^
1	tap water	0.0	2.53 × 10^–2^
2	deionized water	0.0	0.0
3	saline water (35 g L^–1^ NaCl)	18.0	0.0
4	saturated saline (360 g L^–1^ NaCl)	184.7	0.0
5	produced water (55 g L^–1^ NaCl)	41.5 [39]	2.8 [39]
6	saline aqueous suspension (500 g L^–1^ NaCl)	184.7	0.0

Previous studies have shown that increasing the ionic strength of the aqueous phase can enhance W/O emulsion stability by modifying interfacial interactions.^47,49–51^ In crude oil systems, this effect is associated with reduced electrostatic attraction between droplets and increased adsorption of resins and asphaltenes at the interface.^49^ However, such stabilization is not unlimited, and excessive ionic strength may lead to interfacial film disruption.

In the present model systems, droplet size distribution analysis (Figure 4) suggests that Emulsions 3 and 5 are expected to exhibit enhanced kinetic stability owing to their comparatively smaller volume-weighted mean diameters. In both systems, *D* ^3,4^ decreased from 16.71 to 9.09 μm (Emulsion 3) and from 16.39 to 7.06 μm (Emulsion 5), respectively (Figure 4). However, despite their similar droplet size evolution, gravitational separation measurements revealed that only Emulsion 5 maintained kinetic stability for up to 24 h (Figure 4). This discrepancy indicates that the droplet size alone does not fully determine the macroscopic stability. The improved resistance to phase separation observed for Emulsion 5 can be attributed to its significantly higher Ca^2+^ concentration (2.8 g L^–1^) compared with the much lower level found in tap water (2.53 × 10^–2^ g L^–1^), indicating that the higher concentration of the divalent cation enhances interfacial structuring and retards coalescence, in agreement with previous reports.^29,52^

The reduction in droplet size observed in Emulsions 3 and 5 (Figure 4), which contain Na^+^ (E3 and E5) and Ca^2+^ (E5) ions in the aqueous phase, indicates the predominance of the salt-in effect, whereby the solubility of the surfactant (comprising both hydrophilic and hydrophobic groups) is favored by the increase in the ionic strength of the solution.^52^ Additionally, the presence of salt reduces the electrostatic repulsion between the surfactant head groups, promoting a denser and more efficient packing of molecules at the oil–water interface.^53^ From this perspective, the presence of these salts increases the activity of the surfactants at the interface for the kinetic stabilization of these emulsions since the increased solubility and reduced repulsion allow more surfactant molecules to accumulate at the interface, increasing the energy barrier against coalescence and slowing the destabilization of the system toward coalescence.

Additionally, according to Pearson’s hard and soft acids and bases theory, the ether oxygen (ROR group) can be classified as a hard base, whereas Na^+^ and Ca^2+^ cations are considered hard acids.^54^ Consequently, this hard–hard interaction is energetically favored. The potential ion–dipole interactions between the Na^+^ or Ca^2+^ ions and the ethoxylated chains of Triton X-114 can attract the polar head groups of the surfactant, thereby enhancing its adsorption at the oil–water interface. This could promote the formation of a denser interfacial layer, increasing steric repulsion and consequently improving emulsion stability. Since the complexation of divalent cations is stronger than that of monovalent cations for resins and asphaltenes, as reported in the literature,^49,55,56^ a similar trend may occur in the present system. This observation may also explain the greater stabilization observed in emulsions prepared with produced water.

Other factors should also be considered when evaluating the relationship between ionic strength and emulsion stability. The medium lubricating oil that composes the oily phase has a certain degree of polarity because of its acidity of 0.605 mg KOH g^–1^. The polar components responsible for this acidity are most likely attracted to the cations present in the aqueous phase. This interaction would bring the polar compounds closer to the interface, and through this attraction, they can compress the droplets, resulting in a decrease in droplet size and ensuring greater stability. The influence of oil acidity increases the stability of Emulsion 3, especially Emulsion 5, which presents Ca^2+^ ions and is capable of more pronounced ion–dipole interactions because of its smaller ionic radius and greater charge.

Although Perles et al.^49^ reported that increasing the ionic strength improved the stability of W/O emulsions, the authors also reported a limit. After a certain ionic strength, the emulsions exhibit instability because of the amount of surfactant species at the interface, which can result in film disintegration. The instability caused by high salt concentrations is observed in emulsions with relatively high concentrations of Na^+^ ions, which are Emulsions 4 and 6. These emulsions had greater mean droplet sizes and separated water volumes, as shown in Figures 3 and 4.

At high Na^+^ concentrations, a salting-out effect occurs, where the increased ion concentration in the aqueous phase of the emulsion reduces the solubility of nonelectrolyte compounds (surfactants).^52^ This behavior occurs because of the competition of ions for solvation water, which reduces the ability of water molecules to interact with the polar groups of surfactants, such as the ethoxylated chains of TX-114, and decreases the affinity of surfactants for the aqueous phase. This reduction in surfactant solvation can alter its packing at the water–oil interface of the emulsions, thereby reducing the thickness of the steric layer. The reorganization of surfactants at the interface tends to decrease the elasticity and mechanical strength of the film, facilitating liquid film drainage during the approach of droplets in the higher-salinity emulsions studied.

When the aqueous saline suspension (500 g L^–1^ NaCl; Emulsion 6) was compared with the saturated saline water (360 g L^–1^ NaCl; Emulsion 4), greater phase separation was observed for the saturated solution. Although the droplet size increased over time for the saline aqueous suspension, a pronounced difference in macroscopic separation was evident. Since the salting-out effect is expected to be similar in both highly saline systems, the improved stability of the suspension is likely associated with the presence of fine particulates, which contribute to enhanced dispersion stability compared with that of the saturated solution.

The composition of tap water includes several alkaline earth cations, such as Ca^2+^ and Mg^2+^.^38^ The greater stability of the emulsion with tap water (emulsion 1) than that of the emulsion with deionized water (emulsion 2) can be attributed to the presence of these cations. In this case, the interaction of Ca^2+^ with the surfactant and with the polar compounds of the oil did not result in smaller average droplet sizes for Emulsion 1, since the values of the average droplet sizes are similar for both emulsions (Figure 4). This behavior is assumed to be due to the low concentration of Ca^2+^ ions (2.53 × 10^–2^ g L^–1^) compared with the concentration in the produced water (2.8 g L^–1^). Nevertheless, even at low concentrations, divalent cations can influence interfacial structuring through the salting-in effect. Such modifications may alter droplet–droplet interaction forces and retard film drainage during droplet approach, thereby reducing coalescence and subsequent phase separation.

Interactions between anions and water molecules occur primarily through hydrogen bonding and contribute to the overall structuring of the aqueous phase, thereby influencing interfacial behavior and droplet–droplet interactions. Although the ionic strength of each aqueous phase was not explicitly calculated, qualitative differences can be inferred from their ionic compositions. Tap water and deionized water represent the lowest ionic strength systems because of their minimal dissolved ion content. In contrast, the saturated solution and saline aqueous suspension NaCl correspond to the highest ionic strength conditions, reflecting their elevated Na^+^ concentrations. The 35 g L^–1^ saline solution and produced water (55 g L^–1^ NaCl) are expected to exhibit intermediate ionic strength values relative to these extremes. This qualitative ranking is consistent with the observed stability trends, suggesting the existence of an optimal ionic strength range that enhances interfacial organization and kinetic stability, whereas excessively high ionic strength promotes destabilization.

The effect of salinity observed in the present model systems contrasts with the findings reported by Silva et al.^9^ for W/O petroleum emulsions. In their study, no significant phase separation was detected over time, and the most stable system corresponded to the emulsion prepared with deionized water (10% v/v aqueous phase, 3 min of agitation at 2500 rpm), which also presented the smallest mean droplet size. Petroleum emulsions are primarily stabilized by complex interfacial films formed by resins and asphaltenes, which assemble into structured, viscoelastic layers at the oil–water interface. These aggregates differ fundamentally from the relatively small and well-defined nonionic surfactant molecules (TX-114) used in the present systems. In crude oil emulsions, large interfacial aggregates may influence film rheology, reducing droplet size. In contrast, in model systems stabilized by low-molecular-weight surfactants, ionic effects more directly modulate surfactant packing and interfacial organization, leading to different salinity–stability relationships.

The proposed model for the kinetic stability of the six studied emulsions is shown in Figure 5, which correlates the salinity with the surfactant adsorption density at the oil–water interface. For the emulsions with intermediate salinity (produced water—E5 and 35 g L^–1^ saline solution—E3), the highest surfactant density at the interface is observed. In the case of E5, the presence of divalent cations (Ca^2+^) increases the salting-in effect, in addition to interacting with the ethoxylated groups of the surfactant to form a compact interfacial film, which decreases coalescence. For the emulsion with lower salinity (deionized water—E2), moderate surfactant packing is observed, in which the hydration of the polar head groups is maintained without interference from high ionic concentrations. E1 (tap water) corresponds to the baseline for comparison with relative stability because of the presence of divalent ions. Under saturated and saline aqueous suspension conditions, excess Na^+^ ions trigger the salting-out effect. This phenomenon decreases the solubility of the surfactant in the aqueous phase, reducing its affinity for the interface and resulting in a fragile film. In Emulsion 6, the presence of NaCl crystals (gray square) in the bulk aqueous phase confirms the system saturation, without contributing to interfacial reinforcement, which favors pronounced flocculation and an increase in droplet size.

**Figure 5:**
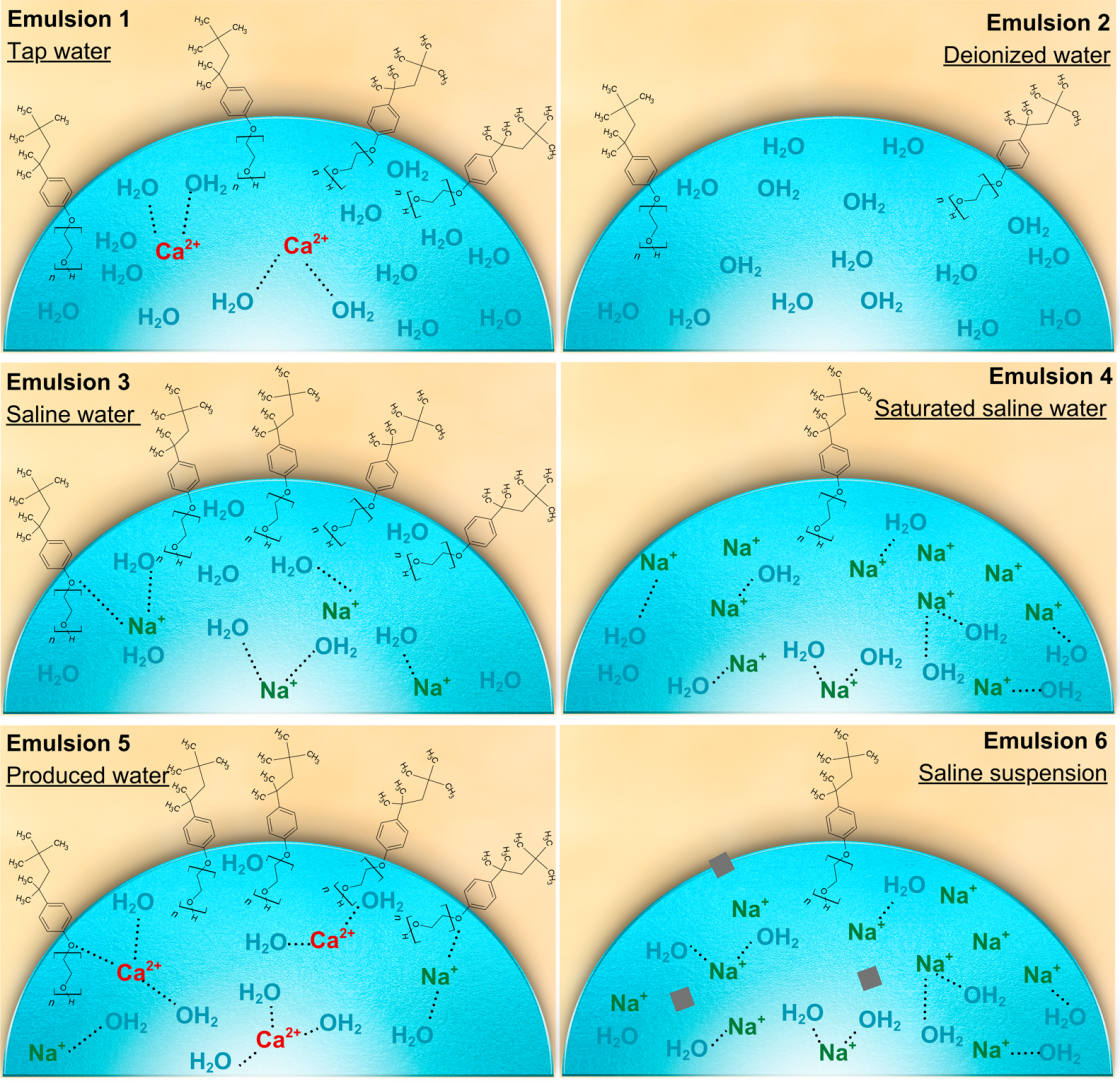
Illustration of the stabilization of the surfactant TX-114 at the emulsion interface under different aqueous phase salinities.

### 3.3 Effect of Salinity on Interfacial Tension

The interfacial tension between the oil phase and each aqueous phase is shown in Figure 6. The interfacial tension (IFT) results show the following behavior: the higher the salinity of the system is, the lower the tension. A reduction in IFT is generally associated with enhanced emulsification, as a lower IFT facilitates droplet breakup during homogenization and increases interfacial area generation.^57^

**Figure 6:**
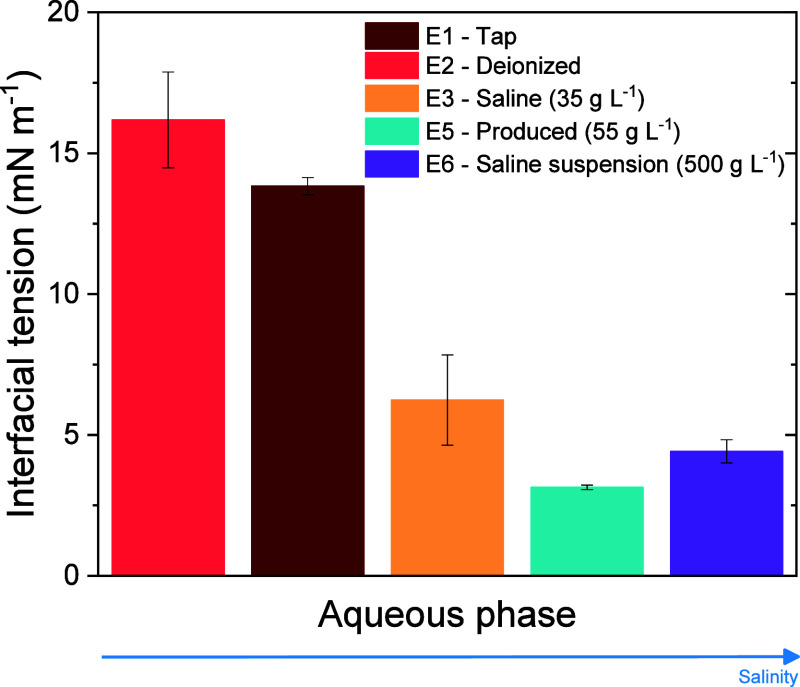
Interfacial tension (IFT) between the medium lubricating oil (containing 0.1% v/v Triton X-114) and the different aqueous phases as a function of salinity, measured by the pendant drop method at 25 °C.

However, the kinetic stability of emulsions is not determined exclusively by interfacial tension. Although low values favor droplet formation, resistance to instability phenomena such as coalescence depends primarily on the properties of the surfactant film formed at the interface, as previously described in Section 3.2.

The lower interfacial tension between the produced water (E5) and the oil phase is attributed to the presence of divalent ions. This stability is corroborated by the gravitational separation analysis and the droplet size distribution. The interaction follows the logic described in Section 3.2. Thus, the reduction is attributed to the salting-in effect, which enhances the surfactant activity at the interface.^52,58^ Beyond the general contribution of cations, the specific presence of divalent cations in this emulsion is decisive for improved stabilization. This occurs because Ca^2+^ favors the formation of a more robust and organized interfacial film, acting in parallel with the ion–dipole interactions of the polar oil compounds with Ca^2+^ and Na^+^ ions, as well as the interaction of the TX-114 oxygen atoms with these cations. The IFT results of the model oil across different salinities closely resemble those of crude oil. Previous studies^59,60^ have shown that the equilibrium interfacial tensions of crude oil in the presence of divalent cations are lower than those observed with monovalent cations. This parallel behavior validates the model oil’s reliability, ensuring more accurate interfacial interpretations and enhanced operational safety.

The interfacial tension behavior differs from the stability results observed for Emulsion 6 (500 g L^–1^ NaCl). Despite the low IFT, this system is subject to the salting-out effect, where stability is compromised by the reduced hydration of the polar head groups of the surfactant, thereby weakening the interfacial film. Consequently, the pendant drop measurement fails to account for droplet–droplet attraction effects, highlighting the necessity of evaluating all the characterization parameters to select the optimal formulation.

### 3.4 Effects of Salinity on Morphology

The microstructural homogeneity of the emulsions was monitored by optical microscopy at each storage time. As the emulsification process consists of a random agitation method, the emulsion produced is characterized as a polydisperse system in which small and large water droplets coexist. The most appropriate description for an emulsion is its droplet size distribution (DSD).^61^ This distribution provides a statistical survey of the fragmentation of the dispersed phase.^44^

Droplet size distributions were determined by laser diffraction and complemented by morphological observations via optical microscopy. In emulsified systems, distributions weighted toward smaller droplet sizes are generally associated with enhanced kinetic stability because of reduced gravitational separation and slower coalescence processes.^44^

Photomicrographs of Emulsion 4 (saturated saline water) and Emulsion 6 (saline aqueous suspension) revealed pronounced droplet flocculation, indicating reduced stability under high ionic strength conditions (Figure 7). In addition, the images of Emulsion 6 showed precipitation of NaCl crystals within the aqueous phase and, after 24 h, partial localization of solids near the interface.

**Figure 7:**
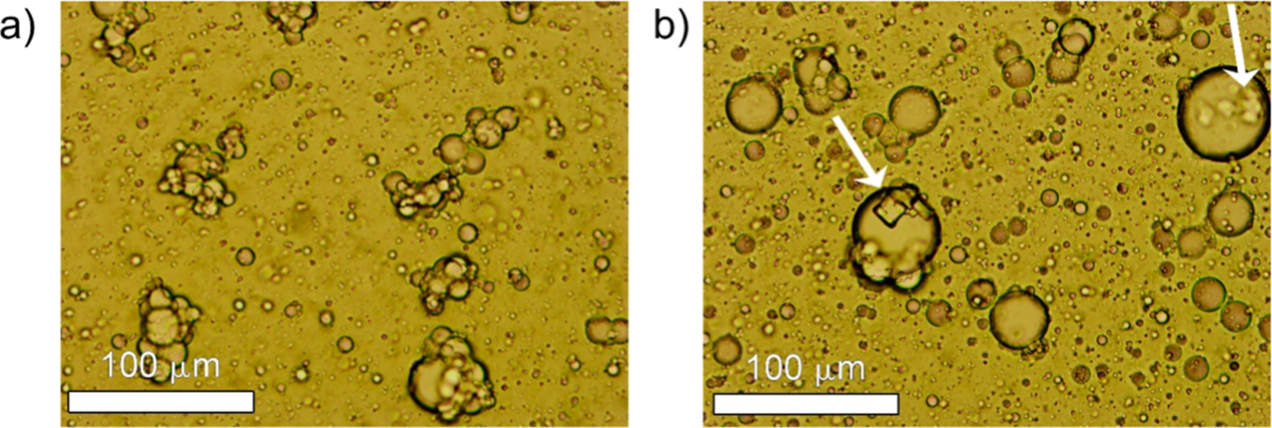
Optical photomicrographs obtained after 24 h of storage: (a) Emulsion 4 prepared with saturated saline water (360 g L^–1^ NaCl) and (b) Emulsion 6 prepared with saline aqueous suspension (500 g L^–1^ NaCl), highlighting droplet flocculation and salt precipitation (indicated by the arrows) under high ionic strength conditions.

These microscopic observations corroborate the discussion presented in Section 3.2. Although the presence of these solid particles could theoretically contribute to interfacial reinforcement, the results show that their effect was insufficient to provide effective steric stabilization or prevent overall destabilization of the system. Visual observations (Figure 7) indicate that the NaCl crystals do not form a continuous protective layer. Together with the macroscopic sedimentation observed in Figure 2, where larger crystals settle and only a small fraction of fine salt particulates remains dispersed within the emulsion, these results suggest an interfacial contribution of the salt particles. Therefore, additional stabilization in the suspension system can be associated with fine particles at the oil–water interface.

Compared with systems with broad or bimodal distributions, emulsions with narrow or monodisperse droplet size distributions generally have greater kinetic stability, even when their mean droplet sizes are similar.^62^ In the present study, none of the six emulsions displayed monodisperse behavior. As shown in Figure 8, the droplet size distributions are broad. A main peak is observed in the range of 5–20 μm, accompanied by a secondary shoulder extending from approximately 40 to 200 μm in all the samples. From a thermodynamic perspective, larger droplets exhibit lower interfacial curvature and reduced Laplace pressure, which favors their persistence and growth at the expense of smaller droplets. This curvature-driven process can progressively shift the distribution toward larger sizes, ultimately promoting emulsion destabilization if coalescence or ripening mechanisms continue.

**Figure 8:**
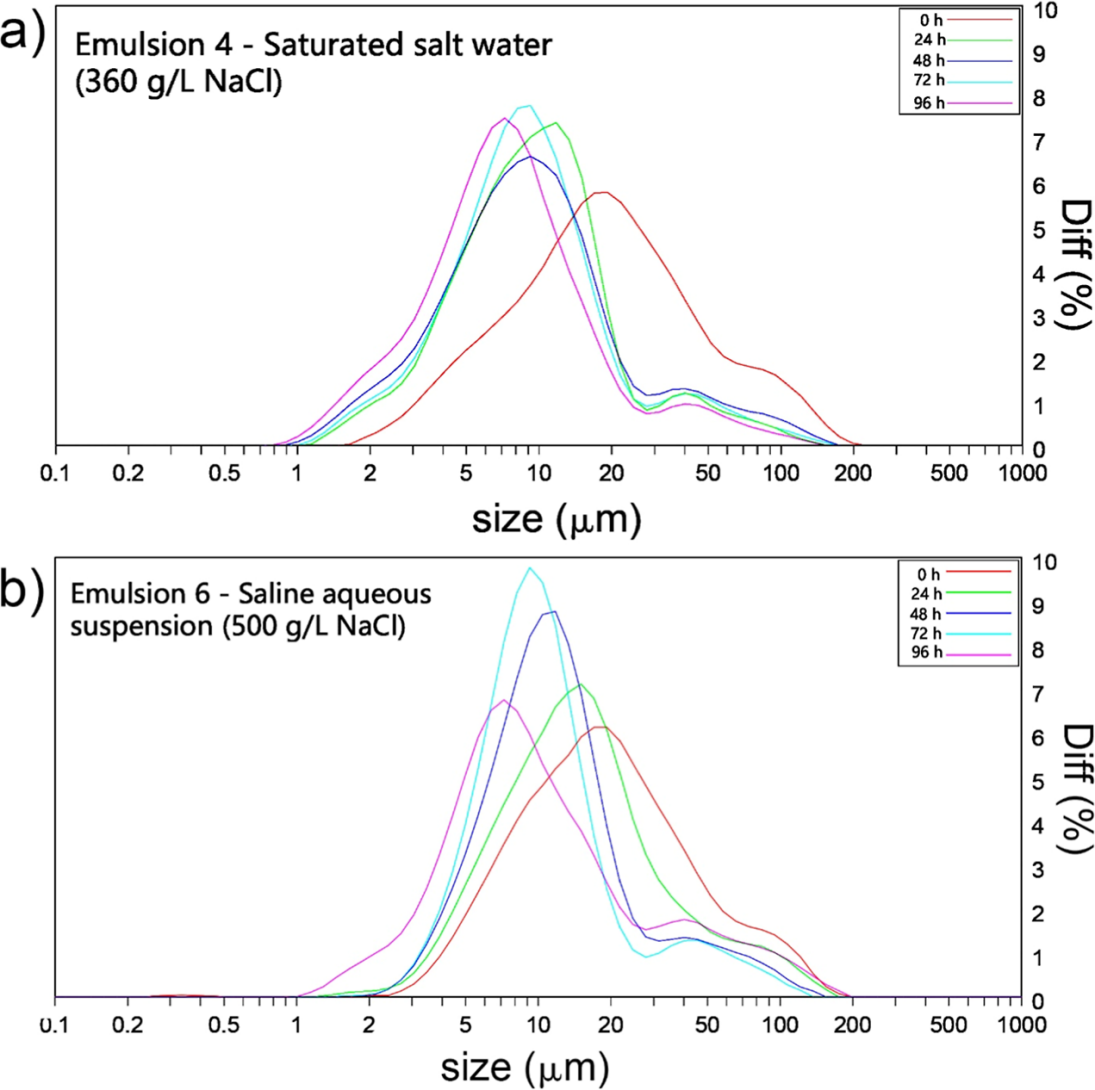
Droplet size distributions (DSDs) obtained via laser diffraction. (a) DSD for Emulsion 4 (saturated saline water, 360 g L^–1^ NaCl), (b) Emulsion 6 (saline aqueous suspension, 500 g L^–1^ NaCl).

A progressive shift in the droplet size distributions toward smaller diameters was observed over the storage time for all the emulsions. This trend is consistent with the photomicrographs, which also show a predominance of smaller droplets at longer time intervals. The observed shift is attributed to gravitational separation and removal of larger droplets through sedimentation, flocculation, and coalescence, resulting in an emulsified phase progressively enriched in smaller droplets.

## 4 Conclusion

This study demonstrated that aqueous phase salinity influences the stability of W/O model emulsions through ionic strength and interfacial organization. While the reduction in IFT facilitated droplet fragmentation, it was not the sole determining factor for long-term stability. In Emulsion 5 (produced water), the presence of divalent cations (Ca^2+^) promoted the salting-in effect, increasing the surfactant activity at the interface and maintaining kinetic stability for up to 24 h. Emulsion 3 (35 g L^–1^ NaCl) exhibited similar droplet size reduction, confirming the beneficial role of moderate ionic strength. These results indicate that salting-in effects and the presence of divalent cations enhance surfactant packing and delay coalescence. This resulted in smaller droplets and a robust interfacial film, which retarded coalescence and phase separation. In contrast, for emulsions prepared with saturated saline solution and saline aqueous suspension (360 and 500 g L^–1^ NaCl), the excessive ion concentration led to a salting-out effect, dehydrating the polar head groups of the surfactants. Despite the low IFT, the weakening of the interfacial film favored flocculation and coalescence, resulting in greater instability. From this perspective, the influence of salinity depends not only on the salt concentration but also on the ion valency (monovalent vs divalent), the type of surfactant and its hydrophilic group, as well as the polar components of the oil phase. Overall, the findings confirm the existence of an optimal salinity window for W/O emulsion stabilization and highlight the importance of jointly considering ionic composition, surfactant behavior, and interfacial structure when designing petroleum-like model emulsions.
